# Drosophila kikkawai – Sox102F

**DOI:** 10.17912/micropub.biology.001211

**Published:** 2024-07-19

**Authors:** Mia Mo, Larissa LoBello, Ismael Hassan Farah, Elwin Agtang, Edith Luz Ramos, Reza Abdoli, Laura Santander Diaz, Larissa Helena Schumann Ferreira, Nighat Kokan, Takrima Sadikot, Alexa Sawa, Cindy Arrigo

**Affiliations:** 1 Washington University in St. Louis, St. Louis, Missouri, United States; 2 Cardinal Stritch University, Milwaukee, Wisconsin, United States; 3 College of the Desert, Palm Desert, California, United States; 4 Washburn University, Topeka, Kansas, United States; 5 Biology, Lakeland University, Plymouth, Wisconsin, United States; 6 Biology, Washburn University, Topeka, Kansas, United States; 7 Biology, College of the Desert, Palm Desert, California, United States; 8 Biology, New Jersey City University, Jersey City, New Jersey, United States

## Abstract

The *Drosophila kikkawai* feature with NCBI Gene ID 108084518 was determined to be an ortholog of *Drosophila melanogaster* *Sox102F* , a member of the FlyBase High Mobility Group Box Transcription Factors gene group (FBgg0000748). Five isoforms were constructed using the GEP F element annotation protocol, the longest being novel isoform Sox102F-PNE (identified using the XM_017180752 RefSeq prediction and RNA-seq data). Among the isoforms found in both *D. melanogaster* and *D. kikkawai* , Sox102F-PB is the longest and exhibits a 1.18x coding span expansion due to transposable element insertion into an intron. All *D. kikkawai* protein isoforms contain the conserved domain HMG_box_dom (IPR009071).

**Figure 1.**
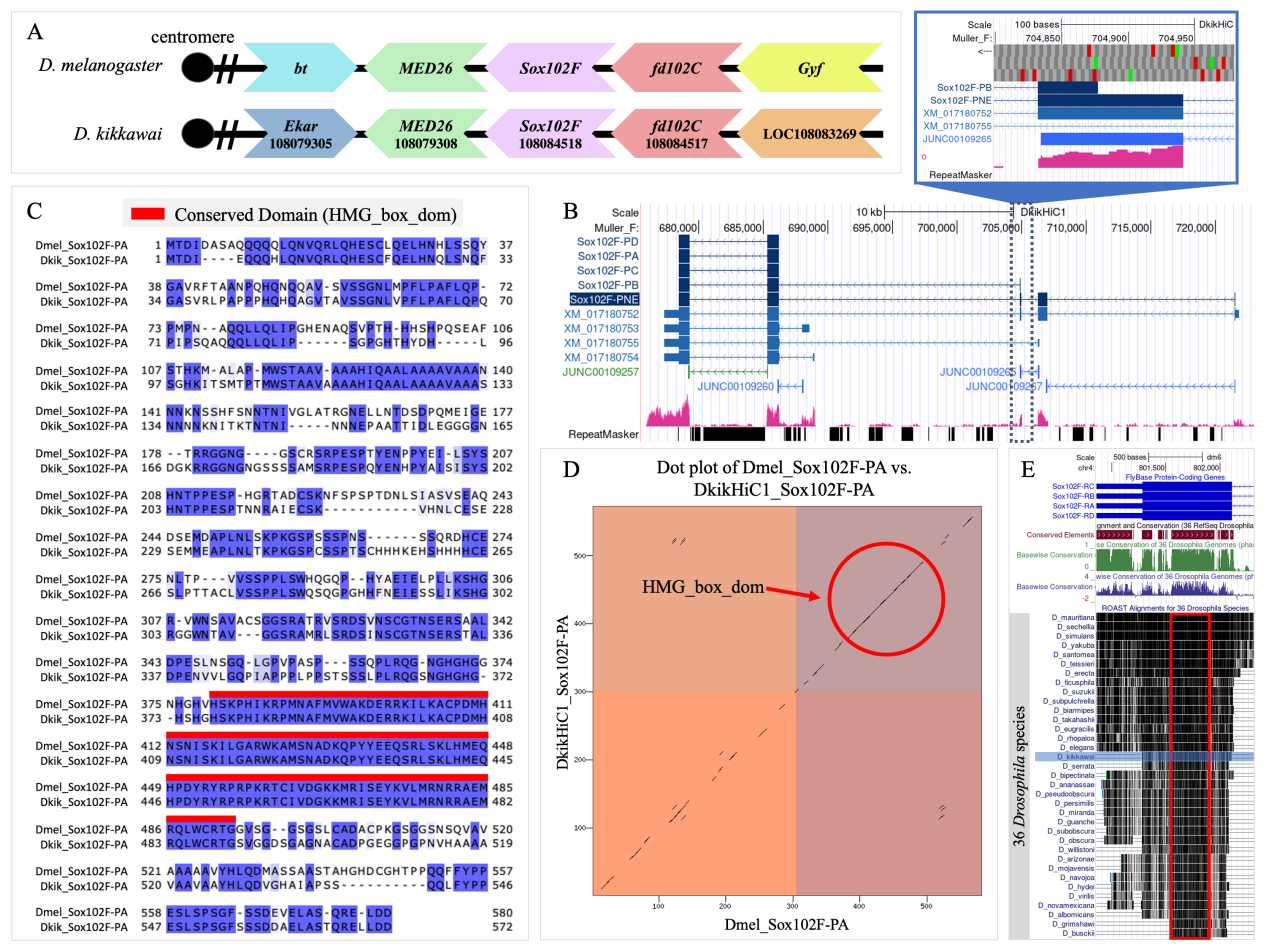
**(A)** **Synteny diagram comparing the gene neighborhood of *Sox102F* in *D. kikkawai* and *D. melanogaster* on the Muller F element.** The large chevron arrows labeled with gene symbols indicate the gene's coding direction relative to the centromere (black dot) *.* For *D. melanogaster* , these labels correspond to the FlyBase gene symbols. For *D. kikkawai* , the labels correspond to the gene symbols in the NCBI Gene database. For *D. kikkawai* genes with published gene symbols, the label begins with the gene symbol (e.g., *Sox102F* ) rather than “LOC”, followed by the NCBI Gene ID (e.g., 108084518). *Sox102F* is coded on the minus strand on the F element in both *D. melanogaster* and *D. kikkawai .* Orthologous genes are denoted with the same color chevron arrow. **(B)** **Genome Browser image of *Sox102F* gene model(s) on the *D. kikkawai* DkikHiC1 assembly with evidence tracks.** The final gene models are labeled with their isoform names and shown in dark blue at the top of the image. Sox102F-PNE is highlighted to indicate the novel isoform in *D. kikkawai* . The provided evidence tracks in order from top to bottom include RefSeq BLAT alignments, RNA-seq coverage from mixed embryos, combined splice junctions, and RepeatMasker (v4.1.2-p1) which displays the locations of different transposable elements (TEs). The inset shows a zoomed in view of the initial Sox102F-PB CDS and an overlapping internal CDS from Sox102F-PNE with a corresponding splice junction and RNA-seq coverage. **(C)** **EMBOSS Needle pairwise alignment output highlighting conserved residues between *D. melanogaster* and *D. kikkawai* Sox102F-PA proteins.** A pairwise alignment output comparing the amino acid (AA) sequence of the *D. melanogaster* Sox102F-PA protein and the resulting AA sequence from the final gene model for Sox102F-PA in *D. kikkawai* . The blue shading demarcates conserved residues with the highly conserved HMG_box_dom (IPR009071) outlined in red. **(D)** **Dot plot depicting protein alignment comparing AA sequences of Sox102F-PA in *D. kikkawai* (y-axis) and *D. melanogaster* (x-axis)** . The abundance of gaps in the diagonal line indicates regions of low sequence similarity. The boxes with alternating colors indicate boundaries between different coding exons. The circled region represents the location of the HMG_box_domain inside the second coding sequence (CDS2) which is shared between *D. kikkawai* and *D. melanogaster* . **(E) ROAST alignment of 36 *Drosophila* species.** The ROAST (release March 2008) alignment depicts conservation across 35 *Drosophila* species against the terminal CDS of all *Sox102F* isoforms in *D. melanogaster* . The darker coloration indicates higher conservation or similarity between species while the light coloration indicates less conservation or similarity. The HMG_box_dom is located within the red boxed region that depicts a stretch of highly conserved sequence across all 36 species including *D. kikkawai* which is highlighted in blue.

## Description

*Drosophila melanogaster* *Sox102F* has been assigned to the High Mobility Group Box Transcription Factors gene family (Pfreundt et al., 2010; Phochanukul & Russell, 2010; Sessa & Bianchi, 2007) . Proteins from this group regulate the *Wnt* signaling pathway and contain a characteristic 80 AA L-shaped DNA minor groove binding domain, which when bound to DNA induces DNA bending. According to FlyBase (release FB2024_02), the *Sox102F* gene is most likely orthologous to either the human *SOX5* or *SOX6* gene, having a DIOPT score of 9/14 when run against both *SOX* genes (Gramates et al., 2022; Hu et al., 2011) . In humans, mutations in the *SOX5* gene are related to Lamb Shaffer Syndrome, a neurodevelopmental disorder (Lamb et al., 2012) . Due to its close association to the brain and development, *Sox102F* in *Drosophila* has been used to study Alzheimer's and heart disease in humans (Li et al., 2013, 2017) . *Drosophila kikkawai* belongs to the *melanogaster* group of the *Sophophora* subgenus (NCBI taxonomy ID: 30033) (Schoch et al., 2020) . This cosmopolitan species is tropical and subtropical, as it is not found above the latitude of 35° (Karan et al., 1998) . *D. kikkawai* is one of four *Drosophila* species (along with *Drosophila takahashii , Drosophila ananassae , Drosophila bipectinata* ) examined in the study of the Muller F element expansion and shows an approximate 1.7-fold increase in chromosome size when compared to the *D. melanogaster* F element (Leung et al., 2023) .

***D. kikkawai* feature with NCBI Gene ID 108084518 is the putative ortholog of *Sox102F*** . The ortholog assignment is supported by a tBLASTn (v2.15.0+; Camacho et al., 2009) alignment using the NCBI BLAST server of the *D. melanogaster* protein sequence for Sox102F-PA (FBpp0088312) against the entire *D. kikkawai* DkikHiC1 (GenBank Assembly Accession: GCA_030179895.1 ) assembly. The top hit maps to scaffold CM058227.1 (assigned to the F element) and reports an E-value of 7e-118, a percent identity of 74.02, and a percent coverage of 99. The coordinates for the top hit (i.e., the match with lowest E-value) correspond to the location of the *D. kikkawai* feature with Gene ID 108084518. The next best hit maps to scaffold CM058225.1 (assigned to the D element) and reports a higher E-value of 8e-25, a lower percent identity of 50.43, and a lower percent coverage of 70. Sox102F-PA is representative of the B, C, D and novel NE isoforms due to the significant CDS overlap among the isoforms. The results of three alignment tools within the genome browser (Spaln, BLAT, tBLASTn) map to the same region which corresponds to the location of the current gene model, providing strong evidence for the ortholog assignment, along with the E-value. Local synteny analysis provides further evidence for ortholog assignment. *Sox102F* is located on chromosome 4 (the F element) in *D. melanogaster* and surrounded by the genes bent ( *bt* ) (FBgn0005666), Mediator complex subunit 26 ( *MED26* ) (FBgn0039923), forkhead domain 102C ( *fd102C* ) (FBgn0039937), and Gigyf ( *Gyf* ) (FBgn0039936). In *D. kikkawai* , the orthologs of Eye-enriched kainate receptor ( *Ekar* ) (FBgn0039916) (Gene ID: 108079305) and Mediator complex subunit 26 ( *MED26* ) (Gene ID: 108079308) are located downstream of the *Sox102F* ortholog while the orthologs of forkhead domain 102C ( *fd102C* ) (Gene ID: 108084517), and *CG31998* (FBgn0051998) (Gene ID: 108083269) are located upstream on the F element. As shown in Figure 1A, the two genes immediately flanking *Sox102F* in *D. kikkawai* are consistent with *D. melanogaster* while the next two genes in the genomic neighborhoods differ between the two species. The *D. kikkawai* feature with the Gene ID 108079305 was determined to be an ortholog of *Ekar* rather than an ortholog of *bt* based on the FlyBase BLASTp (v2.2.18; Altschul et al., 1990) search result of the protein product (XP_041632629) derived from the *D. kikkawai* RefSeq mRNA XM_041776695 against the *D. melanogaster “* Annotated proteins” database. The best BLASTp match is to *D. melanogaster* Ekar-PB with a normalized score of 1555.81 bits and an E-value of 0 (i.e., E-value < 1e-180). The next best hit to CG11155-PD also has an E-value of 0 but a lower score of 969.53 bits. Similarly, the *D. kikkawai* feature with the Gene ID 108083269 was determined to be an ortholog of *CG31998* rather than *Gyf* based on the FlyBase BLASTp search result of the protein product (XP_017034489) derived from the *D. kikkawai* RefSeq mRNA XM_017179000 against the *D. melanogaster* “Annotated proteins” database. The best and only matches are to the A and B isoforms of the *CG31998* gene where the top hit to *D. melanogaster* CG31998-PA reports a normalized score of 1338.94 bits and E-value of 0.

**Characterizing the A, C and D isoforms for *Sox102F*** *.* The *Sox102F* gene is located on the F element of *D. kikkawai .* Isoforms Sox102F-PA, Sox102F-PC, and Sox102F-PD in *D. kikkawai* are conserved relative to the orthologous isoforms in *D. melanogaster* and were annotated according to the protocol described in Rele et al., 2023. In both *D. kikkawai* and *D. melanogaster* , Sox102F-PA (BK067818), Sox102F-PC (BK067819), and Sox102F-PD (BK067820) are comprised of the same two sequences from the unspliced transcript while Sox102F-PB (BK067821), described in further detail below, is comprised of three coding sequences, two shared with the other isoforms and one unique initial CDS ( Figure 1B ). Further analysis of the *Sox102F* feature in *D. kikkawai* led to the discovery of a novel isoform named Sox102F-PNE (BK067822). Nucleotide sequence data reported are available in the Third-Party Annotation Section of the DDBJ/ENA/GenBank databases under the accession numbers TPA: BK067818-BK067822.

**Characterizing Sox102F-PB and novel isoform Sox102F-PNE.** The third CDS of the novel isoform overlaps with the open reading frame of the initial CDS of Sox102F-PB (inset of Figure 1B ). The initial CDS of the Sox102F-PB isoform lacks splice junction support from the combined splice junction track in the GEP UCSC Genome Browser, and the best BLASTx (v2.15.0+) hit does not include the first 6 AA. There are no other nearby in-frame start codons. There were two options to retain the Sox102F-PB isoform, either to modify the gene structure by proposing a novel initial CDS or to truncate the CDS to the nearest start codon at 704,877-704,875. Based on the annotation strategy to construct the most parsimonious gene model compared to the *D. melanogaster* ortholog, the initial CDS for Sox102F-PB was truncated. Due to evidence of splice junctions and RefSeq predictions upstream of this start position, it was concluded that a novel isoform, Sox102F-PNE, whose CDS overlaps that of Sox102F-PB, exists in *D. kikkawai* ( Figure 1B ). Combined splice junctions JUNC00109258, JUNC00109265, and JUNC00109267 mapped to the DkikHiC1 assembly and small RNA-seq peaks from adult males and mixed embryo correspond to the splice boundaries predicted by BLAT (RefSeq mRNA XM_017180752 ), with the latter two junctions scoring reads greater than 10. A combined splice junction score of 10 indicates that the predicted intron is supported by 10 RNA-seq reads, which is the minimum support required for a novel isoform as per protocol. Sox102F-PNE becomes the longest *Sox102F* isoform in *D. kikkawai* . *Sox102F* is involved in the phenomenon known as the F element expansion. The expansion of the *Sox102F* gene was calculated using Sox102F-PB, the longest isoform whose ortholog can be found in *D. melanogaster* . The coding span (from start to stop codon and including introns) of the Sox102F-PB gene in *D. kikkawai* is 26,432 base pairs while its ortholog in *D. melanogaster* has a coding span of 22,317 base pairs. The ~4,000 base pair, or 1.18x, expansion is attributed to the insertion of LINE transposons (TEs) into the intron between Sox102F-PB CDS2 (3_9492_0) and CDS3 (4_9492_0) which are shared across all isoforms. The insertions of these TEs did not alter the gene structure or the predicted amino acid sequence. *D. melanogaster* has no identifiable TEs annotated in the corresponding intron.

**Characterizing HMG_box_domain in *Sox102F*** . As seen in the EMBOSS Needle (v6.6.0.0; Rice et al., 2000) alignment ( Figure 1C ), the HMG_box_domain (IPR009071; Paysan-Lafosse et al., 2023) has been identified in Sox102F-PA and is found to be shared in all isoforms, including the novel NE isoform. This confirms that the feature belongs to the High Mobility Group Box Transcription Factors gene family. Figure 1D depicts that the domain circled in red shows a much higher level of sequence conservation than the rest of the protein when compared to the orthologous *D. melanogaster* protein, alluding to its importance to protein function. Sequence outside of the red circle represent variable regions of lower sequence similarity that do not belong to the conserved domain and vary across species due to the accumulation of mutations over evolutionary time. Across 36 *Drosophila* species the HMG_box_dom is highly conserved in *Sox102F* which can be seen in a ROAST alignment of the terminal CDS ( Figure 1E ). Proteins belonging to the High Mobility Group Box Transcription Factors gene group at FlyBase (FBgg0000748) have been characterized as ubiquitous regulators of development by binding directly to the minor groove of DNA during transcription (Kamachi & Kondoh, 2013; Sessa & Bianchi, 2007) . The *Sox102F* protein's role in development is consistent with the fact that the most abundant subset of supporting RNA-seq coverage is from mixed embryos.

## Methods

The protocol used to annotate and reconcile the *Sox102F* gene model and neighboring gene models can be found in the Rele et al., 2023 paper. The annotations are based on the annotated gene models for FlyBase release FB2022_06 ( *D. melanogaster* release 6.49) in the release 6 assembly (Hoskins et al., 2015) . A mirror of the UCSC Genome Browser (v435) (Kent et al., 2002; Navarro Gonzalez et al., 2021) is maintained by the Genomics Education Partnership (GEP) at https://gander.wustl.edu . Within the *D. kikkawai* Hi-C genome browser, tracks displaying the results of experimental data (e.g., RNA-seq) and computational tools such as tBLASTn (v2.13.0+), Spaln (v2.3.3f), and BLAT (v37x1) were used support the assignment of the *Sox102F* ortholog. The *D. kikkawai* RNA-seq data was generated by the modENCODE project (Chen et al., 2014) . The tBLASTn results report the region of the genome with the highest similarity to *D. melanogaster* protein coding sequences. The Spaln results report the region of the genome with the highest similarity to full-length *D. melanogaster* proteins. BLAT alignments report the region of the genome with the highest similarity to *D. melanogaster* transcripts.

## Extended Data

Description: Transcript, peptide and generic feature format version 3 (GFF3) files for all isoforms (A, B, C, D, NE) of Sox102F for DkikHiC1 assembly. Resource Type: Dataset. DOI: 10.22002/vbjfz-zqn36
